# Different Effects on Protein Expression of CDR132L, an Antisense Inhibitor of miR-132, and Standard Therapies for Myocardial Infarction

**DOI:** 10.3389/fcvm.2022.887236

**Published:** 2022-05-13

**Authors:** Oriol Iborra-Egea, Alberto Aimo, Antoni Bayes-Genis

**Affiliations:** ^1^ICREC (Heart Failure and Cardiac Regeneration) Research Programme, Health Sciences Research Institute Germans Trias i Pujol (IGTP), Barcelona, Spain; ^2^Hospital Universitari Germans Trias i Pujol, Barcelona, Spain; ^3^Institute of Life Sciences, Scuola Superiore Sant'Anna, Pisa, Italy; ^4^Cardiology Division, Fondazione Toscana Gabriele Monasterio, Pisa, Italy; ^5^CIBER Cardiovascular, Instituto de Salud Carlos III, Madrid, Spain

**Keywords:** heart failure, CDR132L, therapy, myocardial infarction, models

## Introduction

Heart failure (HF) development is a common complication of myocardial infarction (MI), which warrants a search for novel therapies able to prevent left ventricular remodeling after an MI. In a recent article, Batkai et al. evaluated CDR132L, a synthetic antisense inhibitor of miR-132, in a pig model of reperfused MI (1). The authors report that monthly intravenous administration of CDR132L is safe and effective in preventing HF development. They expect CDR132L to have an additive, and possibly synergistic, effect to standard-of-care therapies [beta-blockers, angiotensin-converting enzyme inhibitors/angiotensin receptor blockers (ACEi/ARB), and mineralocorticoid receptor antagonists (MRA)] because of their distinct first targets. Nonetheless, a degree of overlap in the final effects of CDR132L and current therapies might exist, given that ACEi/ARB and MRA ultimately modulate myocardial inflammation and fibrosis, as CDR132L do (1).

## Transcriptomic and Bioinformatic Perspective on CDR132L Targeted Therapy

We assessed this point by searching for similar changes in protein expression between MI therapies and CDR132L.

We retrieved the 14 mRNAs significantly altered in the myocardium of pigs receiving CDR132L compared with control pigs: BMPR2, ADRA1D, GCLC, CD44, PRDX1, ECM1, LEP, GATA3, GPX1, EIF4G1, ACE2, HMOX1, RTN4, and LIFR. Except for RTN4, all these mRNAs were downregulated by CDR132L (1). We assumed a close correlation between changes in mRNA levels and the expression of the corresponding proteins, as previously demonstrated (2). By using massive public databases, such as Drugbank (3), the Open Targets Platform (4) and the Human Protein Atlas (5), we identified all approved, investigational and experimental drugs reported to modulate the expression of at least one of these 14 proteins in any setting (Table 1).

**Table 1:** All drugs/compounds that target one or more of the proteins encoded by the 14 mRNAs candidates.

**Protein identifier**	**Protein name**	**# drugs targeting the protein**	**Drug name**	**Effect**
ADRA1D	Adrenoceptor alpha 5 1D	25	Dapiprazole Tamsulosin Methotrimeprazine Doxazosin Terazosin Alfuzosin Dronedarone Silodosin Prazosin Nicardipine Amitriptyline Nortriptyline Imipramine Doxepin Epinephrine Carvedilol Fenoldopam Cabergoline Methoxamine Phenoxybenzamine Phentolamine Quinidine Verapamil Racepinephrine Pizotifen	Downregulation
ACE2	Angiotensin I converting enzyme (peptidyl-dipeptidase A) 2	4	SPP1148 N-(2-Aminoethyl)-1-aziridineethanamine Chloroquine Hydroxychloroquine	Downregulation
PRDX1	Peroxiredoxin 1	3	Copper Zinc Artenimol	Downregulation
BMPR2	Bone morphogenetic protein receptor, type II	2	Dibotermin alfa Fostamatinib	Downregulation
CD44	CD44 molecule	2	Hyaluronic acid Bivatuzumab	Downregulation
GCLC	Glutamate-cysteine ligase	1	Cysteine	Downregulation
GATA3	GATA binding protein 3	1	Pyrrothiogatain	Downregulation
ECM1	Extracellular matrix protein 1	0	–	–
LEP	Leptin	0	–	–
GPX1	Glutathione peroxidase 1	0	–	–
EIF4G1	Eukaryotic translation initiation factor 4 gamma 1	0	–	–
HMOX1	Heme oxygenase 9 (decycling) 1	0	–	–
LIFR	Leukemia inhibitory factor receptor alpha	0	–	–
RTN4	Reticulon 4	0	–	–

## Discussion

We did not find any drug modulating more than one of the 14 proteins at the same time. Therefore, no drug, including ACEi/ARB or MRA, proved able to mimic the effects of CDR132L on protein expression. This finding corroborates the conclusion that CDR132L might have an additive or synergistic action to standard drugs, given the different effects on the profiles of protein expression.

Next, we performed a protein-protein interaction analysis to know if the candidates were biologically related to each other. Then, by using unsupervised algorithms (K-means clustering, elbow method K = 4) we wanted to assess if these interactions corresponded to proteins grouped in the same cluster (and thus share similar biological properties or pathways) or are among proteins from distinct clusters that could indicate more complex biological mechanisms at play. Here we found that HMOX1, GPX1, GCLC, and PRDX1 work to tightly regulate endothelial cell proliferation [false discovery rate (FDR) = 0.002] and hydrogen peroxide catabolic processes (FDR = 0.001). Although we could not find any report on novel drugs or compounds acting to modulate this specific cluster (or the individual proteins), this analysis indicates that a drug targeting them could be highly specific and a possible novel treatment in HF.

## Author Contributions

OI-E and AA contributed to conception and design of the study. OI-E performed the *in silico* analysis. AA wrote the first draft of the manuscript. OI-E, AA, and AB-G wrote sections of the manuscript. AB-G supervised the study. All authors contributed to manuscript revision, read, and approved the submitted version.

## Funding

This work was supported in part by grants from MICINN (PID2019-110137RB-I00 and PLEC2021-008194), Instituto de Salud Carlos III (PIC18/00014, ICI19/00039, ICI20/00135, PI21/01700, and PI21/01703), Red RICORS (PI21/01703), CIBERCV (CB16/11/00403) as a part of the Plan Nacional de I + D + I, and it was co-funded by ISCIII-Subdirección General de Evaluación y el Fondo Europeo de Desarrollo Regional (FEDER) and AGAUR (2017-SGR-483 and 2019PROD00122).

## Conflict of Interest

The authors declare that the research was conducted in the absence of any commercial or financial relationships that could be construed as a potential conflict of interest.

## Publisher's Note

All claims expressed in this article are solely those of the authors and do not necessarily represent those of their affiliated organizations, or those of the publisher, the editors and the reviewers. Any product that may be evaluated in this article, or claim that may be made by its manufacturer, is not guaranteed or endorsed by the publisher.
